# Predictive value of hyperhidrosis for the incidence of type 2 diabetes mellitus: A population‐based cohort study in Taiwan

**DOI:** 10.1002/kjm2.12796

**Published:** 2023-12-26

**Authors:** Chih‐Kai Wong, Ray C. Hsiao, Mu‐Hong Chen, Cheng‐Fang Yen

**Affiliations:** ^1^ Department of Dermatology MacKay Memorial Hospital Taipei Taiwan; ^2^ Department of Psychiatry, Children's Hospital and Department of Psychiatry and Behavioral Sciences University of Washington Seattle Washington USA; ^3^ School of Medicine National Yang Ming Chiao Tung University Taipei Taiwan; ^4^ Department of Psychiatry Taipei Veterans General Hospital Taipei Taiwan; ^5^ Department of Psychiatry School of Medicine College of Medicine, Kaohsiung Medical University Kaohsiung Taiwan; ^6^ Department of Psychiatry Kaohsiung Medical University Hospital Kaohsiung Taiwan

Hyperhidrosis is thought to stem from the impaired negative feedback mechanism to the hypothalamus, causing the overactivation of the sympathetic nervous system (SNS) and neurogenic hyperexcitability of the reflex circuits; then sweat glands of the body are hyperstimulated and sweat more than what is needed to cool the body.^1^ Review studies have demonstrated that SNS overactivation increases the risk of metabolic syndrome, obesity, and insulin resistance.^2^, ^3^ Whether patients with hyperhidrosis have a higher risk of type 2 diabetes mellitus (T2DM) merits in‐depth study.

We used the National Health Insurance Research Database of Taiwan (applied number: H108128) to investigate the temporal association between hyperhidrosis and T2DM. We included 702 patients (305 males and 397 females) aged ≥18 years with hyperhidrosis (*International Classification of Diseases, 9th Revision, Clinical Modification* [*ICD‐9‐CM*] codes: 705.2, 780.8) diagnosed by dermatologists at least twice between January 1, 1998, and December 31, 2012 and with no history of DM (*ICD‐9‐CM* code: 250) and 7020 age‐, sex‐, income‐, residence‐, and comorbidity‐matched controls (3050 males and 3970 females). Patients were followed up until the diagnosis of T2DM (ICD‐9‐CM codes: 250.*x*0 and 250.*x*2, *x* = 0–9), death, or December 31, 2013. A diagnosis of T2DM was documented by board‐certified internal medicine physicians, endocrinologists, and family medicine physicians based on clinical judgment and a laboratory examination during the follow‐up period. The T2DM diagnosis was existent within 3 months after the laboratory examination of fasting glucose or HbA1c was done. Cox regression models with full adjustment for demographic characteristics, comorbidities, Charlson Comorbidity Index (CCI) scores, and all‐cause clinical visits were employed to analyze the associations between hyperhidrosis, T2DM, and T2DM‐related hospitalization by computing the adjusted hazard ratios (aHRs) and their corresponding 95% confidence intervals (CIs).

This study included a total of 702 patients with hyperhidrosis and 7020 control participants (Table 1). Age, sex, monthly premium, urbanization level of residence, and comorbidities were matched between the groups. The hyperhidrosis group had higher CCI scores and all‐cause clinical visits compared with the control group. During the follow‐up period, 38 (6.75 per 1000 person‐years) patients with hyperhidrosis and 160 (2.79 per 1000 person‐years) participants without hyperhidrosis developed T2DM (*p* < 0.001). The results of Cox regression models revealed that patients with hyperhidrosis had a 2.44‐fold (95% CI: 1.70–3.49) greater hazard of T2DM compared with the control group. The significant association between hyperhidrosis and the hazard of T2DM was found in both men (aHR = 3.42, 95% CI: 2.11–5.53) and women (aHR = 3.00, 95% CI: 1.84–4.87). The hyperhidrosis group who were enrolled at the age of 30–39 (aHR = 2.44, 95% CI: 1.20–4.93) and of ≥40 (aHR = 2.42, 95% CI: 1.53–3.83) were more likely to have the diagnosis of T2DM compared with the control group. Moreover, patients with hyperhidrosis also had a 5.02‐fold (95% CI: 2.03–12.42) greater hazard of T2DM‐related hospitalization compared with those in the control group. The significant association between hyperhidrosis and the hazard of T2DM‐related hospitalization was found in women (aHR = 11.37, 95% CI: 2.74–47.16) but not in men (aHR = 3.24, 95% CI: 0.81–12.92). The hyperhidrosis group who were enrolled at the age of 30–39 (aHR = 12.17, 95% CI: 1.81–81.93) and of ≥40 (aHR = 4.08, 95% CI: 1.11–15.06) were more likely to have T2DM‐related hospitalization compared with the control group.

**TABLE 1:** Demographic characteristics, medical comorbidities, and T2DM incidence in patients with hyperhidrosis and control group.

	Patients with hyperhidrosis (*n* = 702)	Control group (*n* = 7020)	*p*‐Value
Age at enrollment (years, SD)	31.69 (10.64)	31.69 (10.63)	0.996
Sex (*n*, %)			>0.999
Male	305 (43.4)	3050 (43.4)	
Female	397 (56.6)	3970 (56.6)	
Level of urbanization (*n*, %)			>0.999
1 (most urbanized)	253 (36.0)	2530 (36.0)	
2	157 (22.4)	1570 (22.4)	
3 (most rural)	292 (41.6)	2920 (41.6)	
Income‐related insured amount			>0.999
≤15,840 NTD/month	180 (25.6)	1800 (25.6)	
15,841–25,000 NTD/month	312 (44.5)	3120 (44.5)	
≥25,001 NTD/month	210 (29.9)	2100 (29.9)	
Medical comorbidities (*n*, %)			
Hypertension	62 (8.8)	620 (8.8)	>0.999
Dyslipidemia	55 (7.8)	550 (7.8)	>0.999
Obesity	20 (2.8)	200 (2.8)	>0.999
Depressive disorder	43 (6.1)	430 (6.1)	>0.999
Alcohol use disorder	13 (1.9)	130 (1.9)	>0.999
Substance use disorder	16 (2.3)	160 (2.3)	>0.999
Smoking	13 (1.9)	130 (1.9)	>0.999
Atopic dermatitis	35 (5.0)	350 (5.0)	>0.999
Psoriasis	2 (0.3)	20 (0.3)	>0.999
CCI score (SD)	1.14 (1.33)	0.86 (1.24)	<0.001
All‐cause clinical visits (times per year, SD)	12.90 (15.41)	7.76 (11.75)	<0.001
Incidence of T2DM (*n*, 1000 person‐years)	38 (6.75)	160 (2.79)	<0.001
Age at T2DM diagnosis (years, SD)	50.94 (9.71)	51.82 (9.97)	0.672
Duration between enrollment and T2DM (years, SD)	6.82 (3.93)	8.08 (3.62)	0.060
T2DM‐related hospitalization (*n*, 1000 person‐years)	9 (1.60)	13 (0.23)	<0.001

Abbreviations: CCI, Charlson Comorbidity Index; NTD, new Taiwan dollar; SD, standard deviation; T2DM, type 2 diabetes mellitus.

SNS overactivation increases catecholamine release and overstimulates sweat gland secretion.^4^, ^5^ Moreover, SNS activation has substantial metabolic effects by several ways.^2^ First, liver releases glucose if the hepatic sympathetic nerves are stimulated. Second, the stimulation of nerves supplying the pancreas reduced the production of insulin and then increased the level of glucagon in portal blood. Third, activation of sympathetic fibers innervating adipose tissue leads to lipolysis and peripheral arterioles constriction, then the chance of impaired glucose uptake in skeletal muscle increases. The hyperhidrosis group had higher all‐cause clinical visits compared with the control group in this study; it raises the possibility that patients with hyperhidrosis are likely to be detected earlier with T2DM compared with the comparison group. Physicians should routinely monitor blood sugar levels and assess symptoms of T2DM in patients with hyperhidrosis to ensure early detection.

## CONFLICT OF INTEREST STATEMENT

All authors declare no conflict of interest.

## IRB APPROVAL STATUS

This study was approved by the Institutional Review Board of Taipei Veterans General Hospital (2018‐07‐016AC).

## References

[1] Nawrocki S , Cha J . The etiology, diagnosis, and management of hyperhidrosis: a comprehensive review: etiology and clinical work‐up. J Am Acad Dermatol. 2019;81:657–666. 30710604 10.1016/j.jaad.2018.12.071

[2] Schlaich M , Straznicky N , Lambert E , Lambert G . Metabolic syndrome: a sympathetic disease? Lancet Diabetes Endocrinol. 2015;3:148–157. 24731670 10.1016/S2213-8587(14)70033-6

[3] Reaven GM . Insulin resistance, compensatory hyperinsulinemia, and coronary heart disease: syndrome X revisited. Com Physiol. 2010;1:1169–1197.

[4] Harker M . Psychological sweating: a systematic review focused on aetiology and cutaneous response. Skin Pharmacol Physiol. 2013;26:92–100. 23428634 10.1159/000346930

[5] Shibasaki M , Wilson TE , Crandall CG . Neural control and mechanisms of eccrine sweating during heat stress and exercise. J Appl Physiol. 2006;100:1692–1701. 16614366 10.1152/japplphysiol.01124.2005

